# Update of the Brazilian Clinical Practice Guideline for primary immune thrombocytopenia: methodological process and main changes for diagnosis and treatment in the Brazilian Unified Health System (SUS)

**DOI:** 10.1016/j.htct.2026.108564

**Published:** 2026-10-27

**Authors:** Ludmila Peres Gargano, Tassiane Cristine Santos de Paula, Layssa Andrade Oliveira, Clarisse Lobo, Francine Doty Campoy, Paula Villaça, Ana Clara Kneese Nascimento, José Maria Soares Júnior, Rosângela Maria Gomes, Marta da Cunha Lobo Souto Maior, Rosa Camila Lucchetta

**Affiliations:** a Health Technology Assessment Unit, Hospital Alemão Oswaldo Cruz, São Paulo, Brazil; b Instituto Estadual de Hematologia Arthur de Siqueira Cavalcanti, SES, Rio de Janeiro, Brazil; c Santa Catarina Blood Center (HEMOSC), Blumenau, Santa Catarina, Brazil; d Clinical Hospital of the University of São Paulo Medical School (HCFMUSP), São Paulo, Brazil; e Faculty of Medical Sciences, Santa Casa de São Paulo, São Paulo, Brazil; f Brazilian Ministry of Health's (MoH), Brasília, Brazil

**Keywords:** Purpura, Thrombocytopenic, Idiopathic, Practice Guideline, Public health practice, Health Technology Assessment

## Abstract

**Background:**

Primary immune thrombocytopenia requires diagnostic exclusion and individualized management. Recent changes in the therapeutic landscape and health technology assessment recommendations of Brazil prompted an update of the national Clinical Practice Guidelines for immune thrombocytopenia in the Unified Health System (SUS).

**Objectives:**

This study aims to describe the methodological process used to update the guidelines for primary immune thrombocytopenia, summarize the primary conceptual, diagnostic, and therapeutic updates, and emphasize the integration of health technology assessment into treatment pathways within the Brazilian Unified Health System.

**Methods:**

The update followed the technical guide of the Brazilian Ministry of Health for guideline development. Recommendations were appraised by the National Committee for Health Technology Incorporation (Conitec) and drafted by a multidisciplinary panel including health technology assessment researchers, clinical experts and policymakers. Systematic reviews, economic evaluations, budget impact analyses, stakeholder input and public consultation drafted the final recommendations.

**Results:**

The updated Clinical Practice Guidelines changed the title and conceptual framing to reflect contemporary disease nomenclature and immune‑mediated pathophysiology, reinforced a diagnostic approach based on clinical evaluation and exclusion of alternative causes of thrombocytopenia, and reorganized classification criteria. Rituximab and romiplostim were incorporated for selected adult and pediatric populations with refractory, chronic or corticosteroid‑dependent disease, whereas dapsone was not incorporated. Treatment sequencing was clarified, positioning immunosuppressive therapies before thrombopoietin receptor agonists and specifying age‑related indications. A public consultation refined the wording and interpretation of the therapeutic sequencing, particularly for pediatric care.

**Conclusion:**

The updated Brazilian Clinical Practice Guidelines for primary immune thrombocytopenia modernizes disease framing, expands treatment options and improves organization of therapeutic decision‑making in the Unified Health System (SUS), illustrating how health technology assessment outputs can be integrated into evidence‑informed national clinical protocols.

## Introduction

Primary immune thrombocytopenia (ITP) is an acquired autoimmune disorder characterized by a transient or persistent reduction in platelet counts, impaired megakaryocyte function, and increased risk of bleeding. The primary form is defined by isolated thrombocytopenia in the absence of other identifiable causes, whereas secondary ITP refers to thrombocytopenia associated with underlying conditions such as systemic autoimmune diseases, malignancies, chronic viral infections, or drug exposure [1,2].

The clinical presentation of ITP is highly heterogeneous. Patients may be asymptomatic or present with petechiae, ecchymoses, hematomas, mucosal bleeding, fatigue, or, less frequently, severe gastrointestinal, genitourinary, or intracranial bleeding. Although lower platelet counts correlate with an increased risk of hemorrhage, the relationship between platelet count and clinical severity remains inconsistent, and patients with equivalent platelet counts often exhibit markedly different bleeding phenotypes. This variability makes clinical assessment central to decisions on observation, treatment initiation and monitoring [3,4].

ITP may occur at any age, but pediatric and adult presentations differ clinically and prognostically. In children, especially those younger than ten years of age, ITP is commonly acute, self-limited, and associated with a high probability of spontaneous remission, while adults more often experience chronic or relapsing disease [2,5]. Approximately 80% of pediatric cases resolve within 6 to 12 months even without specific treatment, whereas adult ITP frequently becomes chronic [6,7]. These differences justify explicit age-sensitive recommendations for diagnosis, treatment selection, and follow-up.

The management of ITP has evolved substantially over the last decade. Beyond first-line corticosteroid-based treatment, the therapeutic armamentarium for patients with persistent, chronic, refractory, or corticosteroid-dependent disease has expanded, including immunosuppressive agents, biologic therapies, and thrombopoietin receptor agonists (TPO-RAs) [8]. These developments have challenged older therapeutic algorithms and increased the need for updated, structured, and context-specific guidance for public health systems. In this context, international clinical guidelines have progressively incorporated emerging scientific evidence to inform clinical decision-making. These recommendations emphasize the need for individualized treatment strategies that consider bleeding risk, patient characteristics, and patient preferences, while also highlighting the importance of minimizing prolonged corticosteroid exposure and promoting patient-centered approaches to care [9, 10, 11, 12].

In Brazil, Clinical Practice Guidelines (CPG) are official documents of the Ministry of Health (MoH) designed to support the organization of care and to standardize diagnostic and therapeutic pathways within the Unified Health System (*Sistema Único de Saúde*, SUS) [8]. These documents are intended to provide evidence-informed recommendations for diagnosis, treatment, monitoring, and regulation of access to healthcare technologies, and are nationally applicable across states and municipalities. The present article aims to describe the methodological process used to update the Brazilian CPG for ITP and to summarize the main conceptual, diagnostic, and therapeutic changes incorporated into the new version of the national guidelines.

## Methods

### Study design

This study is a descriptive methodological report of the update process of the Brazilian CPG for ITP and of the main recommendations of the final document. The article focuses on how the national guidelines were updated in response to new evidence, clinical expert input, public consultation, and health technology assessment (HTA) decisions related to rituximab, romiplostim, and dapsone (technologies prioritized for full HTA process in this update). The AGREE II Reporting Checklist [13] was used to support reporting guideline recommendations (Supplementary material, Table S1).

### Institutional framework

The update was coordinated by the General Coordination of Clinical Protocols and Therapeutic Guidelines (CGPCDT) within the Department of Management and Incorporation of Health Technologies (DGITS), under the Secretariat of Science, Technology, and Innovation in Health (SCTIE) of the Brazilian MoH. The development process followed the MoH methodological guidelines [14], for developing the CPG, which are aligned with the GRADE guidelines [15], including evidence appraisal, formulation of recommendations, National Committee for Health Technology Incorporation (Conitec) appraisal, and public consultation.

The guideline development group included methodological researchers from the Health Technology Assessment Unit (UATS) of Hospital Alemão Oswaldo Cruz (HAOC), clinical specialists with expertise in hematology, pediatric hematology, obstetrics, and related areas, and technical representatives from the MoH. Methodologists supported evidence mapping, review of prioritized technologies, and preparation of HTA materials. Clinical specialists contributed to scope definition, revision of clinical content, research question formulation, interpretation of evidence, and drafting of disease-specific recommendations. MoH representatives ensured alignment with SUS policy, regulation, and implementation requirements.

### Updating process

The update began with a review of the previous CPG, the mapping of recent evidence and international recommendations, and identification of sections requiring revision. Virtual meetings were held between July and August 2023, including pre-scope, scope, and prioritization meetings [16]. During these meetings, the development group discussed the need to change the title of the CPG, revise diagnostic, monitoring and treatment sections, update epidemiological and clinical content, and identify technologies requiring formal assessment for incorporation or non-incorporation decisions.

The development group distinguished between recommendations involving technologies already available in the SUS and technologies requiring formal HTA. Established diagnostic, treatment, or follow-up practices already available in the SUS were revised based on clinical consensus, contemporary evidence, and alignment with current practice, without necessarily generating new research questions. Technologies not yet listed for relevant indication, technologies with uncertainty regarding positioning, and technologies requiring incorporation decisions were prioritized for structured research questions and Conitec assessment.

### Health technology assessment process and research questions

During the scoping process, rituximab, dapsone, and romiplostim were prioritized for the full HTA process because they represented clinically relevant second-line options for patients with refractory, chronic, or corticosteroid-dependent ITP and required explicit decisions on SUS coverage. Two research questions were structured for refractory ITP, one for adults and the other for children and adolescents.

The HTA process included systematic evidence synthesis, assessment of certainty of evidence, an economic evaluation, budget impact analysis, patient perspectives, acceptability, feasibility, implementation considerations, and Conitec deliberation. All analyses were conducted from the perspective of SUS, evaluating the place in therapy of each technology within the overall care pathway rather than assessing individual interventions in isolation.

### Public consultation and final approval

The draft CPG was reviewed by the Technical Subcommittee for Clinical Protocols and Therapeutic Guidelines and by the Conitec CPG Committee, which, at the 141st Conitec meeting on June 6, 2025, recommended its submission to public consultation with a preliminary favorable recommendation for publication. The Public Consultation No. 52/2025, held from June 24 to July 14, 2025, received 236 contributions, mainly from patients and family members, friends, or caregivers. These contributions were read, categorized, and qualitatively analyzed to refine the final version of the protocol. At the 144th Conitec meeting on September 5, 2025, Conitec unanimously recommended updating the CPG for ITP, which was subsequently approved and made publicly available through Joint Ordinance of the Secretariat of Specialized Health Care SAES/SCTIE/MS No. 41/2026.

Figure 1 summarizes the integrated process linking the CPG update and the HTA pathway [14,17].

**Figure 1:**
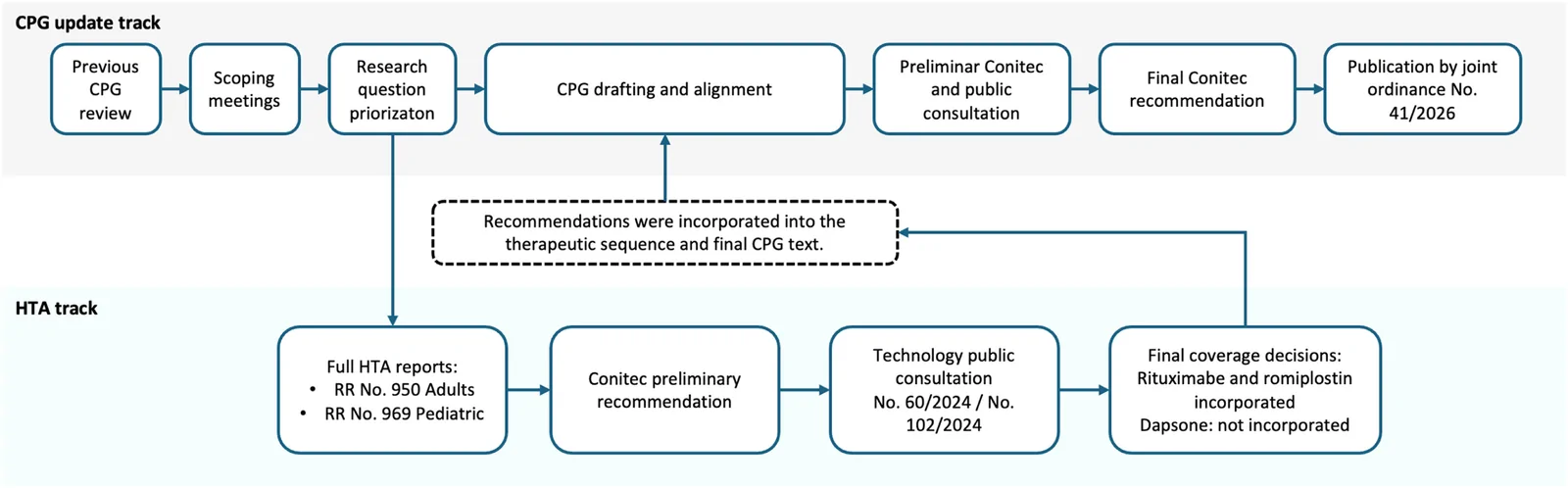
Development and decision pathway for the updated Brazilian Clinical Practice Guidelines (CPG) for primary immune thrombocytopenia. The figure summarizes the CPG update process and the HTA pathway. The update began with review of the previous CPG, scoping meetings, and prioritization of the Population (P), Intervention (I), Comparator (C), and Outcome (O) questions. Prioritized technologies were assessed through full health technology assessment (HTA) reports, followed by Conitec preliminary deliberations, public consultations, and final coverage decisions. These HTA recommendations were then incorporated into the therapeutic sequence and final text, which underwent CPG public consultation, final Conitec recommendation, and publication by joint ordinance SAES/SCTIE. RR: recommendation report.

## Recommendations

### Overview of major changes

The updated CPG introduced conceptual, diagnostic, therapeutic, monitoring and operational changes [18]. The CPG is intended for healthcare professionals involved in ITP care, as well as patients, family members, and caregivers seeking guidance on diagnosis, treatment, monitoring, and follow-up within the SUS nationwide.

The most visible conceptual change was the title update from “Idiopathic Thrombocytopenic Purpura” to “Primary Immune Thrombocytopenia”, aligning the guidelines with the contemporary understanding of immune-mediated mechanisms and acknowledging that purpura is not present in all patients [19]. The diagnostic section was reorganized to emphasize exclusion of secondary causes and non-immune thrombocytopenia, including pseudothrombocytopenia, liver disease, bone marrow failure, inherited thrombocytopenias, infections, autoimmune diseases, pregnancy-related thrombocytopenia, and thrombotic microangiopathies.

Therapeutically, the update expanded the list of medicines available in the SUS for ITP by incorporating rituximab and romiplostim for selected populations [20,21]. It also clarified that observation remains appropriate for many children and clinically stable adults, that corticosteroids should be used for the shortest feasible duration, and that second-line therapies should be sequenced based on response, contraindication, safety, cost-effectiveness, and feasibility. Table 1 summarizes the main changes incorporated into the updated CPG.

**Table 1:** Main changes introduced in the updated Brazilian Clinical Practice Guidelines (CPG) for primary immune thrombocytopenia (ITP).

Domain	Previous or unresolved issue	Updated approach in the CPG
Disease nomenclature	Older title used “Idiopathic Thrombocytopenic Purpura”	Title changed to Primary Immune Thrombocytopenia, preserving the acronym ‘*PTI*’ in Portuguese and better reflecting immune-mediated pathophysiology
Diagnostic framing	Need for clearer distinction between primary and secondary causes	Diagnosis defined as isolated platelet count below 100 × 10^3^/µL after exclusion of other causes of thrombocytopenia
HTA integration	Technologies used or discussed in practice required formal positioning	Rituximab, dapsone, and romiplostim were prioritized for HTA in adults and in children/adolescents
SUS medicine list	Rituximab and romiplostim were not previously positioned in the CPG for the updated indication	Rituximab and romiplostim incorporated for adults and for children/adolescents according to Conitec decisions; dapsone not incorporated [20,21]
Treatment sequence	Need to clarify the place of immunosuppressive agents and TPO-RAs	Azathioprine, cyclophosphamide, or rituximab are positioned before eltrombopag or romiplostim; the CPG does not require all patients to receive rituximab before a TPO-RA
Pediatric treatment	Public consultation raised concerns about rituximab and TPO-RA sequencing in children	Final text clarified that rituximab is an option within the immunosuppressive step and that at least one option among azathioprine, cyclophosphamide, or rituximab should be used before TPO-RAs unless contraindicated
Monitoring	Need for clearer pretreatment and follow-up requirements	Added or clarified screening for hepatitis B, hepatitis C, HIV, active and latent tuberculosis, immunoglobulins before rituximab, pregnancy testing for selected drugs, and monitoring for TPO-RA-related marrow changes
Emergency care	Need to clarify options when corticosteroids and IVIg are insufficient	Vincristine was added as a possible alternative in selected emergencies, especially when rapid response is needed or first-line emergency measures fail

HTA: Health technology assessment; SUS: Brazilian Unified Health System; TPO-RA: thrombopoietin receptor agonists; IVIg: intravenous immunoglobulin.

### Diagnosis and classification

The updated CPG reinforces that ITP remains a diagnosis based on the exclusion, with no specific confirmatory laboratory test. Routine antiplatelet antibody testing is not recommended because of limited diagnostic accuracy. Diagnostic evaluation should be based on clinical and family history, physical examination, complete blood count, platelet count, and systematic exclusion of alternative causes of thrombocytopenia [22]. Physical examination should assess bleeding manifestations and signs suggestive of other conditions, such as lymphadenopathy or hepatosplenomegaly [23]. Peripheral blood smear assessment is essential to identify platelet aggregates, morphological abnormalities, inherited platelet disorders, pseudothrombocytopenia, or other hematological diagnoses [24]; when available, collection in a non-EDTA anticoagulant tube, such as sodium citrate, is recommended to exclude EDTA-induced pseudothrombocytopenia.

Additional tests should be guided by age and clinical suspicion. In adults, anti-human immunodeficiency virus (HIV) and anti-hepatitis C virus testing should be routinely requested, while *Helicobacter pylori* testing may be considered in chronic ITP or in patients with gastrointestinal symptoms [11]. Liver and renal function tests, antinuclear antibodies, protein electrophoresis, and immunoglobulin levels may be useful when autoimmune, lymphoproliferative, or immunodeficiency conditions are suspected. Bone marrow evaluation is not required for initial diagnosis and should be reserved for atypical presentations, such as suspected malignancy or myelodysplasia, additional cytopenias, dysplasia on blood smear, lack of response to ITP therapy, or suspected platelet production abnormalities [11,25].

ITP should be classified by disease phase and treatment response. Newly diagnosed ITP refers to disease duration shorter than three months from diagnosis; persistent ITP refers to duration from three to 12 months; and chronic ITP refers to duration longer than 12 months. Partial response is defined as a platelet count between 30 × 10^3^/µL and 100 × 10^3^/µL or doubling of baseline platelet count without clinically relevant bleeding. Complete response is defined as platelet count of at least 100 × 10^3^/µL without clinically relevant bleeding. Therapeutic failure is defined as platelet count below 30 × 10^3^/µL or less than twice baseline, with persistent or worsening bleeding symptoms. Refractory ITP is defined by a persistent low platelet count despite appropriate use of first-line therapies considered safe for the patient [24,26].

### Inclusion and exclusion criteria

Patients of any age may be included in the CPG if they have ITP, defined as platelet count below 100 × 10^3^/µL after exclusion of secondary or alternative causes of thrombocytopenia, including infections, autoimmune diseases, immunodeficiencies, malignancies, adverse drug reactions, inherited thrombocytopenias, pregnancy-related thrombocytopenia, and other clinically relevant conditions.

Eligibility for pharmacological treatment depends on bleeding severity, platelet count, bleeding risk factors, disease duration, previous treatment response, corticosteroid dependence, and contraindications. First-line treatment (prednisone, dexamethasone, methylprednisolone, or IVIg) is indicated for clinically significant bleeding, platelet count below 30 × 10^3^/µL, or platelet count below 50 × 10^3^/µL with bleeding or relevant risk factors. Subsequent treatment lines (including azathioprine, cyclophosphamide, rituximab, eltrombopag or romiplostim) require persistent or chronic ITP and lack of response, contraindication, or corticosteroid dependence after prior therapies, while treatment-specific exclusions include intolerance, hypersensitivity, or contraindications to the recommended interventions (Figure 2).

**Figure 2:**
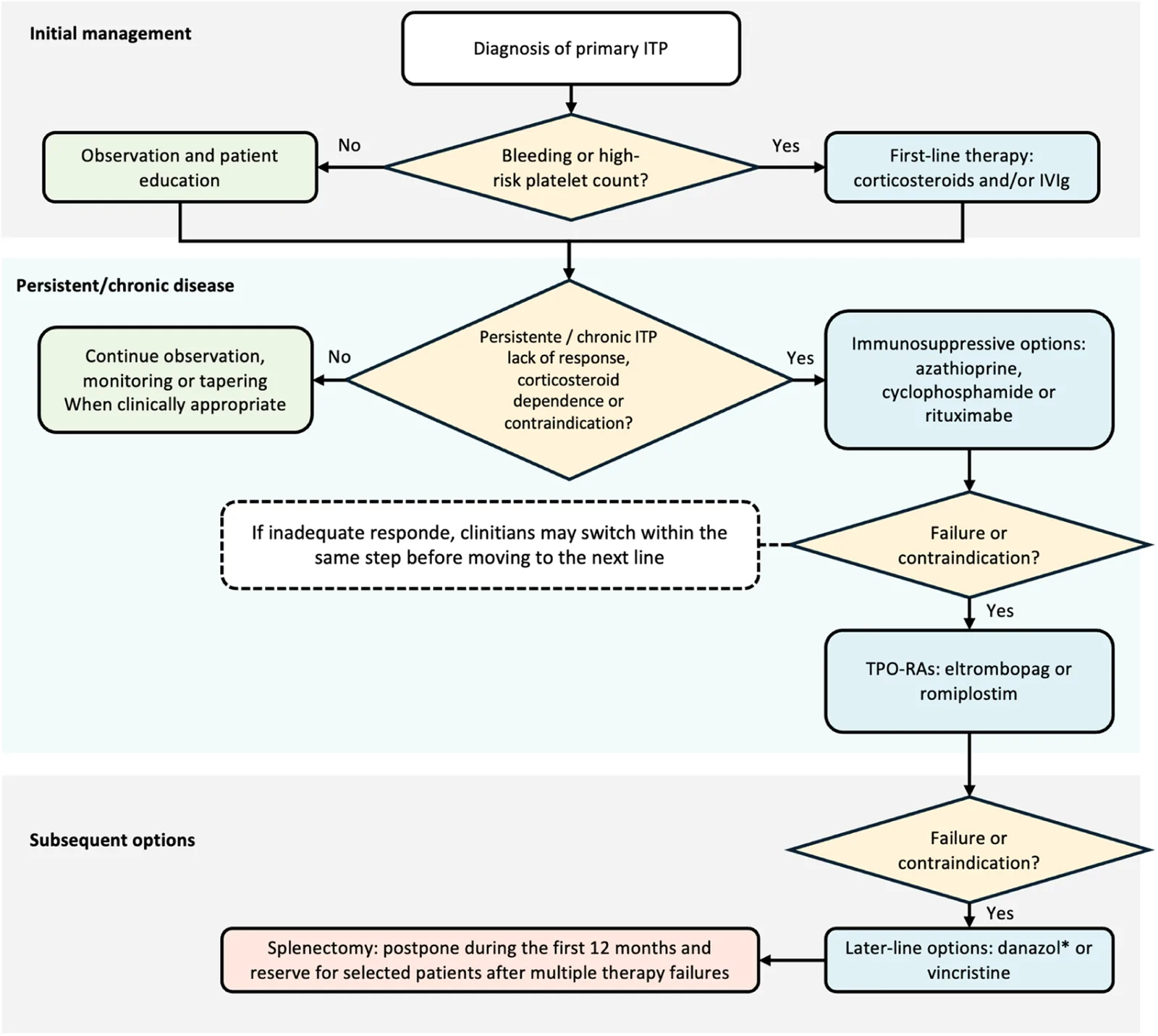
Therapeutic flowchart for primary immune thrombocytopenia (ITP) in the Brazilian Unified Health System (SUS) according to the updated Clinical Practice Guidelines (CPG). Rituximab is indicated for patients over two years of age; eltrombopag is indicated for patients over six years of age; romiplostim is indicated for patients over one year of age; and danazol is not recommended for use by children under 18 years of age. ITP: immune thrombocytopenia; IVIg: intravenous immunoglobulin; TPO-RA: thrombopoietin receptor agonists.

### Observation and treatment initiation

Not all patients with ITP require immediate pharmacological treatment. Observation with clinical and laboratory monitoring is appropriate for children with acute disease limited to cutaneous manifestations, such as petechiae and hematomas, and for those without evidence of clinically relevant bleeding [27, 28, 29, 30]. This recommendation reflects the high likelihood of spontaneous remission in children and the need to avoid unnecessary treatment toxicity.

Observation is also an option for adults with mild to moderate thrombocytopenia, usually platelet counts between 30 × 10^3^/µL and 50 × 10^3^/µL, provided there is no bleeding and no relevant bleeding risk factor [27, 28, 29, 30]. Treatment is recommended for patients with platelet counts below 30 × 10^3^/µL, active bleeding, or special situations requiring higher platelet targets. Patients under observation should be instructed about signs of bleeding, avoidance of contact sports when appropriate, and avoidance of drugs with antiplatelet activity such as acetylsalicylic acid and nonsteroidal anti-inflammatory drugs [28,31].

The psychosocial burden of ITP should also be acknowledged. When patients experience relevant psychosocial impact or significant impairment in quality of life, referral for psychological support should be considered. This addition is important because fatigue, anxiety related to bleeding risk, activity restrictions, and uncertainty regarding platelet fluctuations may affect daily functioning even when severe bleeding is absent.

### First-line pharmacological treatment

Corticosteroids and IVIg are first-line pharmacological treatments. Corticosteroids remain the standard initial therapy and may reduce bleeding independently of platelet count increase. However, corticosteroids should be used for the shortest feasible duration because adverse effects can occur rapidly and may outweigh benefits over time. These adverse effects include behavioral changes, sleep disorders, increased appetite, weight gain, edema, osteoporosis, opportunistic infections, gastric symptoms, cataracts, adrenal insufficiency, hypertension, hyperglycemia, and metrorrhagia [32,33].

Prednisone or prednisolone may be used as initial oral corticosteroid therapy. Dexamethasone may be used when a faster platelet increase is needed, particularly in adults with severe thrombocytopenia below 20 × 10^3^/µL who are asymptomatic or have bleeding without significant clinical repercussion. Methylprednisolone may be used intravenously when rapid platelet increase is required, including as an alternative or adjunct to IVIg in more severe bleeding situations [34,35].

In children without major bleeding, it is recommended that prednisone be given for less than seven days to minimize adverse events. Patients who require long-term corticosteroid therapy for more than eight weeks, or repeated corticosteroid cycles, should be referred to specialized services with ITP experience and considered eligible for treatment of refractory disease.

IVIg is also part of first-line treatment and may be used in children and adults [36]. Its effect may occur faster than corticosteroids, sometimes within 1–2 days, with platelet increase reported within the first hours in some patients. Because its response is usually transient, IVIg is particularly useful when rapid platelet recovery is needed, such as clinically relevant mucosal bleeding, digestive or urinary tract bleeding, severe bleeding, or peri‑procedural management. In severe bleeding or high-risk situations, IVIg may be combined with high-dose methylprednisolone or platelet transfusion [37].

### Second-line treatment and updated pharmacological sequence

Patients who remain with persistently low platelet counts, usually below 30 × 10^3^/µL, despite appropriate first-line therapy, or who have corticosteroid dependence or contraindication to first-line medicines, are eligible for second-line treatment. Azathioprine, cyclophosphamide, rituximab, eltrombopag, romiplostim, danazol, and vincristine are available for refractory disease, with age-specific restrictions and positioning.

The recommended sequence reflects clinical experience, safety, availability, and the cost-effectiveness rationale considered in Conitec deliberations (Table 2). For patients with persistent or chronic ITP and lack of response or dependence on first-line treatment, initial second-line therapy should use one of the immunosuppressive options: azathioprine, cyclophosphamide, or rituximab. If there is contraindication, therapeutic failure, or lack of response, the physician may attempt another medicine within the same treatment step before progressing to the next class.

**Table 2:** Pharmacological treatment sequence for primary immune thrombocytopenia (ITP) in the updated Clinical Practice Guidelines (CPG).

Treatment step	Main medicines	Target population and positioning	Key remarks
Observation	No immediate pharmacological treatment	Children with acute cutaneous-only disease or no bleeding; stable adults with mild to moderate thrombocytopenia and no bleeding risk factors	Requires counseling, monitoring, and reassessment if bleeding or risk factors emerge
First-line therapy	Prednisone/ prednisolone, dexamethasone, methylprednisolone, IVIg	Patients with significant bleeding, platelet count below 30 × 10^3^/µL, or platelet count below 50 × 10^3^/µL with bleeding or risk factors	Corticosteroids should be used for the shortest feasible duration; IVIg is useful when rapid response is needed
Second-line immunosuppressive step	Azathioprine, cyclophosphamide, rituximab	Persistent or chronic ITP with lack of response, corticosteroid dependence, or contraindication to first-line therapies	Rituximab was incorporated into the SUS for adults and children/adolescents according to Conitec decisions
Third-line TPO-RA step	Eltrombopag, romiplostim	Persistent or chronic ITP with lack of response or contraindication to at least one immunosuppressive option	Romiplostim was incorporated as an additional TPO-RA option; switching within the class may be considered
Later-line therapy	Danazol, vincristine	Failure, lack of response, or contraindication to TPO-RAs; vincristine also reserved for selected pediatric and emergency situations	Danazol is not recommended for children or adolescents; vincristine has short time to response but limited durability
Surgical option	Splenectomy	Reserved for selected patients after failure of multiple therapies	Should generally be postponed during the first 12 months after diagnosis

SUS: Brazilian Unified Health System; TPO-RA: thrombopoietin receptor agonists; IVIg: intravenous immunoglobulin.

After failure, lack of response, or contraindication to at least one immunosuppressive option, patients should be treated with a TPO-RA, either eltrombopag or romiplostim. Patients who start one TPO-RA may use a second medicine from the same class in case of failure or lack of response before moving to subsequent therapies. This point is clinically important because eltrombopag and romiplostim have distinct pharmacokinetic, pharmacodynamic, administration, and adherence profiles, and lack of response to one TPO-RA does not necessarily imply lack of response to the other.

Danazol is recommended only for adults after therapeutic failure, lack of response, or contraindication to TPO-RAs. It is not recommended for children and adolescents, especially prepubertal patients, because of the potential of virilization and uncertain safety in this age group. Vincristine is reserved for cases of therapeutic failure or refractoriness to other agents, or for prepubertal children and adolescents with refractoriness or failure to immunosuppressive agents and TPO-RAs.

Table 2 presents pharmacological treatment sequence currently available in the CPG, and Supplementary material, Table S2 details the administration regimens for pharmacological treatments.

### Rituximab, romiplostim, and dapsone in the health technology assessment process

Rituximab, romiplostim, and dapsone were the main technologies prioritized for a full HTA during the update of the Brazilian CPG for ITP. Conitec Recommendation Report No. 950(38) evaluated these technologies for adult patients with refractory or corticosteroid-dependent disease. The assessment indicated that rituximab and romiplostim could be considered therapeutic alternatives to eltrombopag, although certainty of evidence was limited by imprecision and heterogeneity. Dapsone was considered a potential alternative to immunosuppressive therapy, but robust clinical evidence was not identified, and national supply concerns limited its suitability. The adult HTA underwent an important change between preliminary and final recommendations. Initially, Conitec recommended incorporation of rituximab and non-incorporation of romiplostim and dapsone. During Public Consultation No 60/2024, 362 contributions were received, and 97.8% were favorable to the incorporation of the technologies. Participants emphasized the need for additional therapeutic alternatives, potential improvements in quality of life, and the relevance of enabling switching between TPO-RAs. Additional analyses considered treatment sequences and updated economic assumptions. In the final recommendation, Conitec unanimously recommended the incorporation of rituximab and romiplostim and the non-incorporation of dapsone for adults, citing favorable cost-effectiveness relationships and expected efficiency gains for the SUS with rituximab and romiplostim [20,38].

For children and adolescents, Recommendation Report No. 969 [39] assessed the same three technologies for refractory, chronic, or corticosteroid-dependent ITP. Romiplostim was considered an alternative to eltrombopag, and rituximab showed superior platelet response compared with vincristine in the identified randomized trial. No pediatric evidence was identified for dapsone, which was therefore not included in the economic analyses. The pediatric economic evaluation suggested that rituximab alone and rituximab plus romiplostim were dominant compared with the current treatment sequence, while romiplostim alone showed additional Quality-Adjusted Life Years (QALYs) with uncertainty in sensitivity analyses. Following public consultation, Conitec recommended the incorporation of rituximab and romiplostim for refractory, chronic, or corticosteroid-dependent ITP in children and adolescents, and the non-incorporation of dapsone citing scarce pediatric evidence and instability in the national supply [21,39].

A summary of criteria from the GRADE Evidence to Decision (EtD) framework used by Conitec to deliberate on the evidence is presented in the Supplementary material, Table S3 (Table 3).

**Table 3:** Summary of health technology prioritized and assessed for coverage decisions.

Population	Technology	Clinical evidence	Economic assessments	Coverage decision
Adults with refractory or corticosteroid-dependent ITP	Rituximab	Evidence from randomized and observational studies suggested rituximab could be considered an alternative to eltrombopag, although certainty was low to very low due to imprecision and heterogeneity.	Additional analyses after public consultation considered treatment sequences and updated assumptions, supporting favorable efficiency for the SUS.	Incorporated for adults with refractory or corticosteroid-dependent ITP, according to the CPG.
	Romiplostim	Indirect comparisons suggested romiplostim could be considered an alternative to eltrombopag, with limited certainty due to imprecision and heterogeneity.	Initial concerns about budget impact were reassessed after public consultation; updated analyses supported favorable cost-effectiveness and expected efficiency gains.	
	Dapsone	Considered a potential alternative to immunosuppressive therapy, but robust clinical evidence was not identified.	Economic relevance was limited by uncertainty in clinical effectiveness and concerns about national supply availability.	Not incorporated for adults.
Children and adolescents with refractory, chronic, or corticosteroid-dependent ITP	Rituximab	Evidence suggested rituximab was effective and safe; in the identified randomized trial, rituximab showed superior platelet response compared with vincristine.	Rituximab alone, and rituximab combined with romiplostim, were dominant compared with the current treatment sequence, with lower costs and higher QALYs in the model.	Incorporated for children and adolescents with refractory, chronic, or corticosteroid-dependent ITP, according to the CPG.
	Romiplostim	Evidence suggested romiplostim was effective and safe and could be considered an alternative to eltrombopag.	Romiplostim alone increased QALYs, with an ICER of R$14,623 per QALY in the base case, although sensitivity analyses indicated uncertainty and possible higher-cost scenarios.	
	Dapsone	No efficacy and safety evidence was identified for the pediatric population.	Not included in pediatric economic analyses because of absence of clinical evidence.	Not incorporated for children and adolescents.

SUS: Brazilian Unified Health System; CPG: Clinical Practice Guidelines; ITP: Primary immune thrombocytopenia; ICER: incremental cost-effectiveness ratio; QALY: quality adjusted life years.

*Further aspects of the GRADE Evidence-to-Decision (EtD) framework, considered in the Recommendations Reports and discussed during Conitec’s meetings are presented in the Supplementary Material.

### Age-specific pharmacological recommendations

The updated CPG explicitly distinguishes treatment by age eligibility and clinical appropriateness. Rituximab is available for adults and for children older than 2 years; eltrombopag for children older than 6 years, adolescents, and adults; and romiplostim for children from 1 year of age, adolescents, and adults. Danazol is restricted to adults, that is, it is not recommended for pediatric use, while vincristine is reserved for therapeutic failure or refractoriness to other agents, in the treatment of children and pre-pubertal adolescents, and may be considered in selected emergency situations.

This age-specific clarification was particularly important after public consultation. Contributors raised concerns that pediatric recommendations could be interpreted as requiring rituximab before TPO-RAs in all children, despite safety concerns such as hypogammaglobulinemia. The final CPG clarified that rituximab is one option among the immunosuppressive therapies, together with azathioprine and cyclophosphamide, and that at least one of these options should be used before starting a TPO-RA, without mandating rituximab for every patient (Figure 2). This preserves the cost-effectiveness-based sequence while allowing individualized decisions according to age, comorbidities, contraindications, adverse events, adherence, route of administration, bleeding risk, and feasibility of monitoring.

### Emergency situations

Emergency is defined as intracranial or mucosal bleeding, including digestive, genitourinary, or respiratory bleeding, associated with hemodynamic or respiratory instability. Critical bleeding also includes bleeding in anatomical sites such as intra-spinal, intraocular, retroperitoneal, pericardial, or intramuscular bleeding with compartment syndrome, as well as ongoing bleeding causing instability or respiratory compromise [40].

Emergency treatment consists of platelet transfusion with or without IVIg and high-dose corticosteroids [41]. Because transfused platelets are rapidly destroyed in ITP, larger-than-usual transfusion volumes may be required. Vincristine may be considered in selected emergencies, especially when rapid response is needed or when corticosteroids and IVIg fail (Supplementary material, Table S4).

### Surgical treatment

Splenectomy remains a potential option for selected patients because it may provide durable response, but its use has declined with the availability of pharmacological alternatives that avoid surgical risks and long-term infectious complications. The updated CPG recommends postponing splenectomy during the first 12 months after diagnosis whenever possible and reserving it for patients who fail multiple therapies [42].

Before splenectomy, patients should receive vaccination against *Streptococcus pneumoniae, Haemophilus influenzae* type b, and *Neisseria meningitidis* serogroup C at least 15 days before surgery. Platelet count should be above 50 × 10^3^/µL, and IVIg or corticosteroids may be used preoperatively to increase platelet counts.

### Pregnancy and neonatal considerations

Management of ITP during pregnancy should be multidisciplinary, involving obstetricians, hematologists, anesthesiologists, and neonatologists. Treatment should be considered when maternal indications are present, usually platelet counts between 20 × 10^3^/µL to 30 × 10^3^/µL, bleeding, or the need to increase platelet counts to safe levels for procedures or delivery.

Low-dose prednisone is the first-line option during pregnancy, adjusted to the minimum dose needed to achieve a hemostatically effective platelet count. IVIg is an alternative when corticosteroids are contraindicated, ineffective, or when bleeding is present [43,44]. For delivery, platelet counts above 50 × 10^3^/µL are ideally maintained, and counts from 70 × 10^3^/µL to 80 × 10^3^/µL are recommended for spinal or epidural anesthesia. The route of delivery should generally follow obstetric indication rather than ITP alone [37,45,46]. Neonatal thrombocytopenia may occur, although severe complications are uncommon; therefore, newborns may require platelet monitoring.

### Monitoring

Monitoring is designed to support clinical decision-making without creating unnecessary barriers to treatment continuity. For all patients, anamnesis and complete blood count are recommended before treatment and every six months. In adults, hepatitis B, hepatitis C, and HIV serology are required before treatment; in children, these tests are optional in refractory or chronic disease.

Pretreatment and follow-up monitoring vary by drug. Rituximab requires hepatitis B screening and serum immunoglobulin assessment because of risks of viral reactivation and hypogammaglobulinemia. Screening for active and latent tuberculosis is recommended before rituximab, azathioprine, and cyclophosphamide, but drug dispensing should not depend on repeated tuberculosis follow-up tests without clinical indication. Azathioprine and cyclophosphamide require monitoring for hematologic and hepatic toxicity, and renal function when relevant. Eltrombopag requires liver enzyme and bilirubin monitoring, while romiplostim dose adjustment is guided by platelet response. For TPO-RAs, leukoerythroblastic changes associated with elevated lactate dehydrogenase rarely suggest bone marrow fibrosis, in which case bone marrow biopsy, treatment interruption, or switching drug class may be considered. Danazol monitoring includes liver tests, lipid profile, and annual abdominal ultrasound, while vincristine requires monitoring for hematologic toxicity and peripheral neuropathy.

## Discussion

The updated Brazilian CPG for ITP modernizes the national guidance by refining disease nomenclature, diagnostic criteria, treatment eligibility, therapeutic sequencing, and monitoring recommendations within the SUS. Because ITP remains a diagnosis of exclusion and has heterogeneous clinical presentation, the updated CPG reinforces a pragmatic diagnostic approach based on clinical evaluation, complete blood count, peripheral blood smear, and exclusion of secondary causes, while avoiding unnecessary invasive procedures such as bone marrow evaluation in typical initial presentations. This diagnostic clarification is important to reduce inappropriate treatment and to support safer use of immunosuppressive agents and thrombopoietin receptor agonists.

International and national ITP guidelines, and previous Brazilian guidance, provide important clinical references but have limitations for the direct adoption in the context of SUS [9, 10, 11, 12]. TPO-RAs are recognized internationally as major second-line options for patients with persistent or chronic ITP who are corticosteroid-dependent or have an inadequate response to initial therapy. Agents such as eltrombopag, romiplostim, and avatrombopag show high platelet response rates and favorable safety profiles, and are often preferred before splenectomy. However, these guidelines generally place multiple second-line therapies at similar levels and do not incorporate SUS-specific HTA decisions, coverage restrictions, or implementation constraints. For this reason, a full formal adaptation was not feasible in the present update; instead, the therapeutic pathway was structured primarily according to Conitec prioritization and national incorporation decisions, while selected recommendations from the previous Brazilian CPG were retained when considered methodologically and contextually appropriate.

A central contribution of this update is the integration of HTA decisions into the therapeutic pathway. The incorporation of rituximab and romiplostim expands the treatment options available in the SUS for patients with refractory, chronic, or corticosteroid-dependent disease. Rituximab is positioned among immunosuppressive options, while romiplostim adds an additional TPO-RA alternative to eltrombopag, allowing greater individualization according to age, prior response, contraindications, route of administration, monitoring feasibility, and bleeding risk. At the same time, the CPG preserves a structured sequence of care, recommending immunosuppressive options before TPO-RAs and reserving later-line treatments such as danazol, vincristine, and splenectomy for selected situations after previous therapeutic failure or contraindication.

The non-incorporation of dapsone illustrates the role of HTA in delimiting access when evidence and implementation conditions are insufficient. Although dapsone has historical use and a low acquisition cost, the absence of robust comparative evidence, lack of pediatric evidence, and concerns about national supply limited its suitability for incorporation. This reinforces that incorporation decisions in the SUS should consider not only potential clinical benefit and price, but also certainty of evidence, feasibility, availability, and the expected efficiency of resource allocation.

The public consultation contributed meaningfully to the final version of the CPG. Stakeholder input helped clarify pediatric sequencing, avoiding interpretation that rituximab would be mandatory in all children before TPO-RAs; strengthened pretreatment and monitoring recommendations, including infection screening before rituximab and safety monitoring for TPO-RAs; and refined recommendations for emergency situations, pregnancy, and psychological support. These contributions improved the clarity, safety, and implementability, showing the value of participatory processes in national guideline development.

Although international and Brazilian ITP guidelines provided valuable background, their recommendations were not directly adapted due to differences in methodological quality, broader availability of therapies, and limited alignment with SUS HTA and access regulations. The present CPG therefore emphasizes a context-specific therapeutic pathway shaped by national HTA appraisals and coverage decisions.

Some limitations remain. The evidence supporting several second-line comparisons is indirect, heterogeneous, or of low certainty, particularly in adults. Long-term comparative evidence on optimal sequencing is limited, and economic results are sensitive to assumptions about dose, treatment duration, prices, market share, and switching patterns. Therefore, successful implementation will require monitoring of real-world use, including access, treatment sequencing, corticosteroid exposure, switching between TPO-RAs, bleeding outcomes, adverse events, quality of life, and budget impact in SUS administrative data. Overall, the updated CPG represents a clinically relevant and policy-oriented advance by aligning evidence, HTA, stakeholder input, and national access regulation for ITP care in Brazil.

## Conclusion

The updated Brazilian CPG for ITP provides a contemporary, evidence-informed, and operationally structured framework for diagnosis, treatment, and monitoring in the SUS. This update modernizes disease nomenclature, reinforces diagnosis by exclusion, clarifies criteria for treatment initiation, limits prolonged corticosteroid exposure, expands second-line options through the incorporation of rituximab and romiplostim, and establishes a sequence that balances clinical benefit, safety, cost-effectiveness, and feasibility.

By integrating Conitec HTA reports, economic evaluations, budget impact analyses, public consultation, and final regulatory decisions into the national guideline, the CPG exemplifies how universal health systems can translate evidence and policy decisions into practical care pathways. Continued monitoring of implementation and real-world outcomes will be essential to assess whether the updated recommendations improve access, reduce unnecessary corticosteroid exposure, support individualized treatment, and improve outcomes for people living with ITP in Brazil.

## Funding source

This guideline updating process was funded through a partnership with the Brazilian Ministry of Health (MoH) via the Unified Health System (SUS) Institutional Development Support Program (PROADI-SUS). This manuscript was prepared by the Health Technology Assessment Unit (UATS) of the Hospital Alemão Oswaldo Cruz (HAOC).

## Declaration of generative AI and AI-assisted technologies in the writing process

During the preparation of this work the authors used IA tools to improve language and readability. After using this tool/service, all authors reviewed and edited the content as needed and take full responsibility for the content of the publication.

## Conflicts of interest

The authors declare that they have no conflicts of interest to disclose.

## Data Availability

The data that support the findings of this study are available from the corresponding author upon reasonable request.

## Appendix Supplementary materials

Image, application 1

## Footnotes

Supplementary material associated with this article can be found in the online version at doi:10.1016/j.htct.2026.108564.
