# Supplementary material for: Update of the Brazilian Clinical Practice Guideline for primary immune thrombocytopenia: methodological process and main changes for diagnosis and treatment in the Brazilian Unified Health System (SUS)
Source: Hematol Transfus Cell Ther. 2026 Oct 27;49(1):108564. doi: 10.1016/j.htct.2026.108564 (PMC13734884; doi:10.1016/j.htct.2026.108564)
Supplement: Supplementary file 1 [file mmc1.docx]

**SUPPLEMENTARY MATERIAL**

**Update of the Brazilian clinical practice guidelines for primary immune thrombocytopenia: methodological process and main changes for diagnosis and treatment in the Brazilian Unified Health System (SUS)**

**Table S1:** AGREE Reporting Checklist 2016

| **Checklist item and description** | **Reporting criteria** | **Page**  **#** | **Comments** |
| --- | --- | --- | --- |
| DOMAIN 1: SCOPE AND PURPOSE | | | |
| 1. OBJECTIVES  Report the overall objective(s) of the guideline. The expected health benefits from the guideline are to be specific to the clinical problem or health topic. | ☒ Health intent(s) (i.e., prevention, screening, diagnosis, treatment, etc.)  ☒ Expected benefit(s) or outcome(s)  ☒ Target(s) (e.g., patient population, society) | Page 7 and Table 1 | Section “3.1. Overview of major changes” |
| 2. QUESTIONS  Report the health question(s) covered by the guideline, particularly for the key recommendations. | ☒ Target population  ☒ Intervention(s) or exposure(s)  ☒ Comparisons (if appropriate)  ☒ Outcome(s)  ☒ Health care setting or context | Table 4 and Table S2 | Research questions for HTA for listing health technologies. |
| 3. POPULATION  Describe the population (i.e., patients, public, etc.) to whom the guideline is meant to apply. | ☒ Target population, sex and age  ☒ Clinical condition (if relevant)  ☒ Severity/stage of disease (if relevant)  ☒ Comorbidities (if relevant)  ☒ Excluded populations (if relevant) | Page 9 | Section “3.3. Inclusion and exclusion criteria” |
| DOMAIN 2: STAKEHOLDER INVOLVEMENT | | | |
| 4. GROUP MEMBERSHIP  Report all individuals who were involved in the development process. This may include members of the steering group, the research team involved in selecting and reviewing/rating the evidence and individuals involved in formulating the final recommendations. | ☐ Name of participant  ☒ Discipline/content expertise (e.g., neurosurgeon, methodologist)  ☒ Institution (e.g., St. Peter’s hospital)  ☐ Geographical location (e.g., Seattle, WA)  ☒ A description of the member’s role in the guideline development group | Pages 4-5 |  |
| 5. TARGET POPULATION  PREFERENCES AND VIEWS  Report how the views and preferences of  the target population were  sought/considered and what the resulting outcomes were. | ☒ Statement of type of strategy used to capture patients’/publics’ views and preferences (e.g., participation in the guideline development group, literature review of values and preferences)  ☒ Methods by which preferences and views were sought (e.g., evidence from literature, surveys, focus groups)  ☒ Outcomes/information gathered on patient/public  information  ☒ How the information gathered was used to inform the guideline development process and/or formation of the recommendations | Pages 4-5 | Social participation (at Conitec’s meeting) and public consultation are the processes used to assess the preferences and perspectives of the target population, both regarding the clinical guideline and the Recommendation Reports for the evaluated technologies. |
| 6. TARGET USERS  Report the target (or intended) users of the guideline. | ☒ The intended guideline audience (e.g. specialists, family physicians, patients, clinical or institutional leaders/administrators)  ☒ How the guideline may be used by its target audience (e.g., to inform clinical decisions, to inform policy, to inform standards of care) | Page 7 and Table 1 |  |
| DOMAIN 3: RIGOUR OF DEVELOPMENT | | | |
| 7. SEARCH METHODS  Report details of the strategy used to search for evidence. | ☒ Named electronic database(s) or evidence source(s) where the search was performed (e.g., MEDLINE, EMBASE, PsychINFO, CINAHL)  ☒ Time periods searched (e.g., January 1, 2004 to March 31, 2008)  ☒ Search terms used (e.g., text words, indexing terms, subheadings  ☒ Full search strategy included (e.g., possibly located in appendix) | Page 5 | Detailed information related to evidence retrieval, economic evaluations, and the GRADE EtD criteria considered by Conitec during plenary discussions for recommending listing health technologies are detailed in the respective Recommendation Reports (RR), which are publicly available on the agency's website. |
| 8. EVIDENCE SELECTION CRITERIA  Report the criteria used to select (i.e., include and exclude) the evidence. Provide rationale, where appropriate. | ☒ Target population (patient, public, etc.) characteristics  ☒ Study design  ☒ Comparisons (if relevant)  ☒ Outcomes  ☒ Language (if relevant)  ☒ Context (if relevant) | Pages 5 and Table S2. |  |
| 9. STRENGTHS & LIMITATIONS OF THE EVIDENCE  Describe the strengths and limitations of  the evidence. Consider from the  perspective of the individual studies and  the body of evidence aggregated across all the studies. Tools exist that can facilitate the reporting of this concept. | ☒ Study design(s) included in body of evidence  ☒ Study methodology limitations (sampling, blinding, allocation concealment, analytical methods)  ☒ Appropriateness/relevance of primary and secondary outcomes considered  ☒ Consistency of results across studies  ☒ Direction of results across studies  ☒ Magnitude of benefit versus magnitude of harm  ☐ Applicability to practice context | Pages 6-7 |  |
| 10. FORMULATION OF  RECOMMENDATIONS  Describe the methods used to formulate  the recommendations and how final  decisions were reached. Specify any areas of disagreement and the methods used to resolve them. | ☐ Recommendation development process (e.g., steps used in modified Delphi technique, voting procedures that were considered)  ☐ Outcomes of the recommendation development process (e.g., extent to which consensus was reached using modified Delphi technique, outcome of voting procedures)  ☐ How the process influenced the recommendations (e.g., results of Delphi technique influence final recommendation, alignment with recommendations and the final vote) | Pages 4-5 and Figure 1 | Detailing of discussions on the Recommendation Reports (RR), which are publicly available on the Conitec's website. For Conitec’s procedures, refer to the relevant legislation. |
| 11. CONSIDERATION OF BENEFITS AND HARMS  Report the health benefits, side effects, and risks that were considered when formulating the recommendations. | ☒ Supporting data and report of benefits  ☒ Supporting data and report of harms/side effects/risks  ☒ Reporting of the balance/trade-off between benefits and harms/side effects/risks  ☒ Recommendations reflect considerations of both benefits and harms/side effects/risks | Pages 4-5 and Table S2 |  |
| 12. LINK BETWEEN  RECOMMENDATIONS AND EVIDENCE  Describe the explicit link between the  recommendations and the evidence on  which they are based. | ☒ How the guideline development group linked and used the evidence to inform recommendations  ☐ Link between each recommendation and key evidence (text description and/or reference list)  ☐ Link between recommendations and evidence summaries and/or evidence tables in the results section of the guideline | Pages 4-5 and Figure 1 |  |
| 13. EXTERNAL REVIEW  Report the methodology used to conduct the external review. | ☒ Purpose and intent of the external review (e.g., to improve quality, gather feedback on draft recommendations, assess applicability and feasibility, disseminate evidence)  ☒ Methods taken to undertake the external review (e.g., rating scale, open-ended questions)  ☒ Description of the external reviewers (e.g., number, type of reviewers, affiliations)  ☒ Outcomes/information gathered from the external review (e.g., summary of key findings)  ☐ How the information gathered was used to inform the guideline development process and/or formation of the recommendations (e.g., guideline panel considered results of review in forming final recommendations) | Pages 6-7 | External review was carried out during public consultations. The contributions received during the public consultation were presented and discussed during Conitec’s meeting, and are available on the agency’s website. |
| 14. UPDATING PROCEDURE  Describe the procedure for updating the guideline. | ☐ A statement that the guideline will be updated  ☐ Explicit time interval or explicit criteria to guide decisions about when an update will occur  ☐ Methodology for the updating procedure |  |  |
| DOMAIN 4: CLARITY OF PRESENTATION | | | |
| 15. SPECIFIC AND UNAMBIGUOUS  RECOMMENDATIONS  Describe which options are appropriate in  which situations and in which population  groups, as informed by the body of evidence. | ☒ A statement of the recommended action  ☒ Intent or purpose of the recommended action (e.g., to improve quality of life, to decrease side effects)  ☒ Relevant population (e.g., patients, public)  ☒ Caveats or qualifying statements, if relevant (e.g., patients or conditions for whom the recommendations would not apply)  ☒ If there is uncertainty about the best care option(s), the uncertainty should be stated in the guideline | Pages 8-17 |  |
| 16. MANAGEMENT OPTIONS  Describe the different options for managing the condition or health issue. | ☒ Description of management options  ☒ Population or clinical situation most appropriate to each option | Pages 9-16 and Figure 1 |  |
| 17. IDENTIFIABLE KEY  RECOMMENDATIONS  Present the key recommendations so that  they are easy to identify. | ☐ Recommendations in a summarized box, typed in bold, underlined, or presented as flow charts or algorithms  ☒ Specific recommendations grouped together in one section | Pages 8-17 |  |
| DOMAIN 5: APPLICABILITY | | | |
| 18. FACILITATORS AND BARRIERS TO APPLICATION  Describe the facilitators and barriers to the guideline’s application. | ☐ Types of facilitators and barriers that were considered  ☒ Methods by which information regarding the facilitators and barriers to implementing recommendations were sought (e.g., feedback from key stakeholders, pilot testing of guidelines before widespread implementation)  ☒ Information/description of the types of facilitators and barriers that emerged from the inquiry (e.g., practitioners have the skills to deliver the recommended care, sufficient equipment is not available to ensure all eligible members of the population receive mammography)  ☒ How the information influenced the guideline development process and/or formation of the recommendations | Pages 12-13 and Table S2 | Facilitators and barriers for implementation was specifically assessed related to the technologies listed. However, considering the organizational structure of the SUS, all recommendations were made in alignment with the capacity for implementation and delivery of healthcare services across the municipal, state, and federal levels. |
| 19. IMPLEMENTATION ADVICE/TOOLS  Provide advice and/or tools on how the recommendations can be applied in practice. | ☐ Additional materials to support the implementation of the guideline in practice. For example:  o Guideline summary documents  o Links to check lists, algorithms  o Links to how-to manuals  o Solutions linked to barrier analysis (see Item 18)  o Tools to capitalize on guideline facilitators (see Item 18)  o Outcome of pilot test and lessons learned |  |  |
| 20. RESOURCE IMPLICATIONS  Describe any potential resource implications of applying the recommendations. | ☒ Types of cost information that were considered (e.g., economic evaluations, drug acquisition costs)  ☒ Methods by which the cost information was sought (e.g., a health economist was part of the guideline development panel, use of health technology assessments for specific drugs, etc.)  ☒ Information/description of the cost information that emerged from the inquiry (e.g., specific drug acquisition costs per treatment course)  ☒ How the information gathered was used to inform the guideline development process and/or formation of the recommendations | Pages 12-13 and Table S2 | Resource implications were assessed only for newly listed technologies, as the recommendations primarily guide the care already provided within the healthcare system. Detailed information can be access at the Recommendation Reports (RR), which are publicly available on the agency's website. |
| 21. MONITORING/ AUDITING CRITERIA  Provide monitoring and/or auditing criteria to measure the application of guideline recommendations. | ☐ Criteria to assess guideline implementation or adherence to recommendations  ☐ Criteria for assessing impact of implementing the recommendations  ☐ Advice on the frequency and interval of measurement  ☐ Operational definitions of how the criteria should be measured |  |  |
| DOMAIN 6: EDITORIAL INDEPENDENCE | | | |
| 22. FUNDING BODY  Report the funding body’s influence on the content of the guideline. | ☒ The name of the funding body or source of funding (or explicit statement of no funding)  ☐ A statement that the funding body did not influence the content of the guideline | Page 18 |  |
| 23. COMPETING INTERESTS  Provide an explicit statement that all group members have declared whether they have any competing interests. | ☐ Types of competing interests considered  ☐ Methods by which potential competing interests were sought  ☒ A description of the competing interests  ☐ How the competing interests influenced the guideline process and development of recommendations | Page 18 |  |

Adapted from Brouwers MC, Kerkvliet K, Spithoff K, on behalf of the AGREE Next Steps Consortium. The AGREE Reporting Checklist: a tool to improve reporting of clinical practice guidelines. BMJ 2016;352:i1152. doi: 10.1136/bmj.i1152.

**Table S2:** Administration regimens for pharmacological treatments in children, adolescents, and adults with primary immune thrombocytopenia

| **Medicines** | **Children and adolescents** | **Adults** |
| --- | --- | --- |
| **First-line treatment** | | |
| Prednisone or prednisolone | 1 to 2 mg/kg/day orally for 4 to 21 days, with gradual dose tapering after adequate therapeutic response; or 4 mg/kg/day orally for three to four days. | 0.5 to 2 mg/kg/day orally for 2 to 4 weeks, until platelet count increases to 30 x 10^3^/µL to 50 x 10^3^/µL, with gradual dose tapering after adequate therapeutic response. The recommended treatment duration is up to eight weeks; however, if necessary, a dose below 0.5 mg/kg/day may be acceptable after this period. |
| Dexamethasone | 0.6 mg/kg/day, maximum 40 mg/day, orally or intravenously, for four days, during four to eight days. | 40 mg/day orally or intravenously for four consecutive days; one to six cycles every 14 to 28 days. |
| Methylprednisolone | 30 mg/kg/day, high dose, intravenously for three days, followed by 20 mg/kg/day on the remaining days; do not exceed 1 g/day for children up to 11 years of age. | 30 mg/kg/day or 1 g/day intravenously for three days. |
| Intravenous human immunoglobulin | 0.8 g/kg/day to 1 g/kg/day as a single dose, which may be repeated if there is no response or until completing 2 g/kg/cycle; or 400 mg/kg/day for five days. | 0.5 g/kg/day to 1 g/kg/day as a single dose, which may be repeated if there is no response or until completing 2 g/kg/cycle; or 400 mg/kg/day for five days. |
| **Second-line treatment** | | |
| Azathioprine | 2 mg/kg/day orally, daily. | 150 mg/day orally, daily. |
| Cyclophosphamide | 50 mg/m² to 100 mg/m² orally, daily. | 50 mg/m² to 100 mg/m² orally, daily. |
| Rituximab | 375 mg/m² per week intravenously for four weeks. | 375 mg/m² per week intravenously for four weeks. |
| Eltrombopag | Initial dose of 50 mg orally once daily. Adjust the dose to achieve platelet count above 50 x 10^3^/µL, up to a maximum dose of 75 mg/day. For patients of Asian ancestry, including those with hepatic impairment, the initial dose should be 25 mg orally once daily. | Initial dose of 50 mg orally once daily. Adjust the dose to achieve platelet count above 50 x 10^3^/µL, up to a maximum dose of 75 mg/day. For patients of Asian ancestry, including those with hepatic impairment, the initial dose should be 25 mg orally once daily. |
| Romiplostim | Initial dose of 1 mcg/kg/week, administered subcutaneously. Subsequent doses should be adjusted to maintain platelet count within the range of 50 x 10^3^/µL to 20 x 10^3^/µL, up to a maximum dose of 10 mcg/kg/week. | Initial dose of 1 mcg/kg/week, administered subcutaneously. Subsequent doses should be adjusted to maintain platelet count within the range of 50 x 10^3^/µL to 200 x 10^3^/µL, up to a maximum dose of 10 mcg/kg/week. |
| Danazol | Not recommended. | 400 mg/day to 800 mg/day orally, daily. |
| Vincristine | 1.0 mg to 2.0 mg intravenously once weekly for up to four weeks. | 1.0 mg to 2.0 mg intravenously once weekly for up to four weeks. |

a. This dose is effective for inducing response in children and may be used for 4 to 21 days, depending on the desired speed of response. Currently, prednisone for less than 7 days is recommended for patients without major bleeding to minimize adverse events.

b. To avoid complications, gradual dose tapering is recommended as soon as platelet count improves, or within up to four weeks in non-responders.

c. Intravenous methylprednisolone administration is compatible with SUS procedure code 03.03.02.001-6, Pulsotherapy I, per application.

d. Reserved for situations requiring rapid platelet increase, as an alternative to or in combination with IVIg, particularly in more severe bleeding.

e. To minimize adverse events with IVIg, the following measures are recommended: pre-hydration 30 minutes before infusion with 0.9% saline, 10 to 20 mL/kg in children; allowing the product to reach room temperature; adequate reconstitution of lyophilized products; monitoring vital signs every 20 to 30 minutes; and slow infusion, especially during the first administrations, using infusion pumps whenever possible. Infusion should start at 0.01 mL/kg/minute, equivalent to 0.5 to 1 mg/kg/minute, and gradually increase every 15 to 30 minutes to 0.02, 0.04, 0.06, and up to 0.08 mL/kg/minute, equivalent to 4 to 8 mg/kg/minute for 5% and 10% products, over 3 to 6 hours. Patients should remain under observation for 30 to 60 minutes after infusion completion.

f. Eltrombopag is recommended for children older than 6 years of age.

g. Intravenous vincristine administration is compatible with SUS procedure code 03.03.02.006-7, Treatment of coagulation defects, purpura, and other hemorrhagic conditions, which is compatible with ICD-10 code D69.3, Idiopathic Thrombocytopenic Purpura.

Abbreviations: ICD-10, International Classification of Diseases, 10th Revision; ITP, immune thrombocytopenia; IVIg, intravenous immunoglobulin; SUS, Brazilian Unified Health System.

**Table S3:** Summary of Finding (SoF) and GRADE Evidence to Decision for technologies assessed for listing

| **Question 1: Are rituximab, dapsone, and romiplostim effective, safe, cost-effective, and economically viable compared with treatments already available in the SUS for adults with primary ITP that is refractory, chronic, or corticosteroid-dependent?^1^** | | | | | |
| --- | --- | --- | --- | --- | --- |
| Background and motivation: Primary ITP may become persistent or chronic and require additional therapies after corticosteroids and/or IVIg. Before this update, the SUS therapeutic sequence included immunosuppressive agents and eltrombopag, but access to rituximab and romiplostim was not standardized for this indication. During the update of the Brazilian CPG, rituximab, dapsone, and romiplostim were prioritized for full HTA because they were considered clinically relevant alternatives for patients with refractory disease, corticosteroid dependence, or failure of previous therapies. The adult assessment was summarized in Conitec Recommendation Report No. 950. | | | | | |
| Patient or population: Adults with primary ITP that is refractory, chronic, or corticosteroid-dependent  Setting: Outpatient and specialized hematology care in the SUS  Intervention: Rituximab, dapsone, or romiplostim  Comparison: Eltrombopag, azathioprine, cyclophosphamide, vincristine, or danazol, according to the therapeutic line and available evidence | | | | | |
| Outcomes | | | Impact | Nº of studies/ design | Certainty of the evidence (GRADE) |
| Platelet response: romiplostim vs eltrombopag | | | Indirect comparison did not show a statistically significant difference between romiplostim and eltrombopag for platelet response. The NMA estimate was RR 1.79, 95% CI 0.52 to 6.13. | 11 / randomized clinical trials | LOW ⨁⨁◯◯ |
| Platelet response: romiplostim vs eltrombopag, observational evidence | | | Observational studies did not show a consistent difference in platelet response between romiplostim and eltrombopag. Reported estimates included RR 1.0889, 95% CI 0.8731 to 1.3580, and RR 0.7841, 95% CI 0.5091 to 1.2077. | 2 / observational | VERY LOW ⨁◯◯◯ |
| Clinically significant bleeding: romiplostim vs eltrombopag | | | No statistically significant difference was observed in clinically significant bleeding. The NMA estimate was RR 0.67, 95% CI 0.14 to 3.27. | 5 / randomized clinical trials | LOW ⨁⨁◯◯ |
| Serious adverse events: romiplostim vs eltrombopag | | | No statistically significant difference was observed in serious adverse events. The GRADE table reported RR 1.06, 95% CI 0.50 to 2.26. | 9 / randomized clinical trials | LOW ⨁⨁◯◯ |
| Platelet response: rituximab vs eltrombopag | | | Indirect comparison did not show a statistically significant difference between rituximab and eltrombopag for platelet response. The NMA estimate was reported as RR 0.22, 95% CI 0.04 to 1.20. | 11 / randomized clinical trials | LOW ⨁⨁◯◯ |
| Clinically significant bleeding: rituximab vs eltrombopag | | | No statistically significant difference was observed in clinically significant bleeding. The NMA estimate was RR 1.04, 95% CI 0.34 to 3.14. | 5 / randomized clinical trials | LOW ⨁⨁◯◯ |
| Serious adverse events: rituximab vs eltrombopag | | | No statistically significant difference was observed in serious adverse events. The NMA estimate was RR 0.42, 95% CI 0.09 to 1.99. | 9 / randomized clinical trials | LOW ⨁⨁◯◯ |
| Platelet response: dapsone vs azathioprine | | | Observational evidence did not show a statistically significant difference between dapsone and azathioprine. Reported estimates were RR 0.9059, 95% CI 0.5692 to 1.4418, and RR 0.6803, 95% CI 0.1356 to 3.4126. | 2 / observational | VERY LOW ⨁◯◯◯ |
| Explanations:  The certainty of evidence was downgraded mainly because of inconsistency and imprecision, particularly wide confidence intervals crossing the null effect. For observational studies, certainty was very low because of serious risk of bias, heterogeneity in outcome definitions, small sample sizes, and limited reporting of follow-up duration. For dapsone, evidence was especially limited and did not provide robust support for incorporation. | | | | | |
| Problem | Adults with refractory, chronic, or corticosteroid-dependent ITP may require prolonged corticosteroid exposure, repeated rescue therapies, or sequential use of immunosuppressive agents and TPO-RAs. This creates clinical burden, risk of bleeding, treatment toxicity, and inequities in access when effective therapies are not incorporated or clearly positioned in the SUS therapeutic pathway. | | | | |
| Benefits and harm | Rituximab and romiplostim were considered clinically relevant alternatives within the therapeutic sequence. The available evidence did not show statistically significant differences between romiplostim or rituximab and eltrombopag for platelet response, clinically significant bleeding, or serious adverse events, although certainty was low. Romiplostim showed quality-of-life improvements compared with standard care in one study, but comparative interpretation was limited. Dapsone had only very low-certainty observational evidence and no robust comparative support. | | | | |
| Resource use | The adult economic assessment included cost-minimization and budget impact analyses. Rituximab showed lower costs compared with eltrombopag across evaluated scenarios. Romiplostim had greater uncertainty because results were sensitive to assumptions about dose and treatment sequence. Dapsone had low acquisition cost, but its economic attractiveness was undermined by weak evidence and supply concerns. After public consultation, additional analyses considering therapeutic sequences suggested that scenarios including rituximab and/or romiplostim could generate efficiency gains for the SUS. | | | | |
| Equity | No formal equity assessment was reported. However, incorporation of rituximab and romiplostim may reduce unequal access to therapies for refractory or corticosteroid-dependent disease, especially for patients who previously depended on local availability, private coverage, or litigation. | | | | |
| Acceptability | Public Consultation No. 60/2024 received 362 contributions, most of which were favorable to incorporation. Stakeholders emphasized the need for additional therapeutic alternatives, improved quality of life, and the possibility of switching between TPO-RAs. Concerns included the cost and administration route of romiplostim, infusion logistics for rituximab, and supply limitations for dapsone. | | | | |
| Feasibility | Rituximab and romiplostim were considered feasible for incorporation when positioned according to the PCDT sequence. Rituximab requires infusion capacity and safety monitoring, while romiplostim requires weekly subcutaneous administration and dose adjustment based on platelet response. Dapsone was not considered feasible for incorporation because of insufficient robust evidence and instability in national supply. | | | | |
| **Question 2: Are rituximab, dapsone, and romiplostim effective, safe, cost-effective, and economically viable compared with treatments already available in the SUS for children and adolescents with primary ITP that is refractory, chronic, or corticosteroid-dependent?^2^** | | | | | |
| Background and motivation: Children and adolescents with ITP often have a self-limited disease course, but a subgroup develops persistent, chronic, refractory, or corticosteroid-dependent disease requiring additional treatment. The update of the Brazilian CPG prioritized rituximab, dapsone, and romiplostim because pediatric therapeutic options in the SUS were limited, especially for patients who failed first-line treatment or required alternatives to prolonged corticosteroid use. Romiplostim was also relevant because eltrombopag is available only from 6 years of age, leaving an unmet need for younger children. The pediatric assessment was summarized in Conitec Recommendation Report No. 969. | | | | | |
| Patient or population: Children and adolescents with primary ITP that is refractory, chronic, or corticosteroid-dependent  Setting: Outpatient and specialized pediatric hematology care in the SUS  Intervention: Rituximab, dapsone, or romiplostim  Comparison: Eltrombopag, azathioprine, cyclophosphamide, or vincristine, according to age eligibility and available evidence | | | | | |
| Outcomes | | | Impact | Nº of studies/ design | Certainty of the evidence (GRADE) |
| Platelet response: romiplostim vs eltrombopag | | | Indirect comparison did not show a statistically significant difference between romiplostim and eltrombopag. The estimate was RR 1.75, 95% CI 0.64 to 4.76. | 5 / randomized clinical trials | MODERATE ⨁⨁⨁◯ |
| Platelet response: romiplostim vs eltrombopag, observational evidence | | | One observational study showed platelet response in 85.7% of patients treated with romiplostim and 75.0% treated with eltrombopag, without statistically significant difference between groups; RR 1.14, 95% CI 0.79 to 1.66. | 1 / observational | MODERATE ⨁⨁⨁◯ |
| Clinically significant bleeding: romiplostim vs eltrombopag | | | Clinically significant bleeding was more frequent with romiplostim than with eltrombopag in the indirect comparison; RR 3.03, 95% CI 1.12 to 8.33. | 4 / randomized clinical trials | MODERATE ⨁⨁⨁◯ |
| Serious adverse events: romiplostim vs eltrombopag | | | No statistically significant difference was observed in serious adverse events; RR 4.17, 95% CI 0.33 to 33.33. | 4 / randomized clinical trials | MODERATE ⨁⨁⨁◯ |
| Platelet response: rituximab vs vincristine | | | Platelet response was higher with rituximab than with vincristine: 69.2% vs 37.5%; RR 1.85, 95% CI 1.04 to 3.29. | 1 / randomized clinical trial | MODERATE ⨁⨁⨁◯ |
| Dapsone for pediatric ITP | | | No efficacy or safety evidence was identified for dapsone in the pediatric population. | No eligible studies | Not assessable |
| Explanations:  For romiplostim versus eltrombopag, the certainty of evidence was rated as moderate, mainly downgraded because of imprecision. No head-to-head randomized trial directly compared these TPO-RAs, so the main comparative estimates came from indirect evidence. For rituximab versus vincristine, certainty was moderate, with risk of bias concerns in the identified randomized trial. Dapsone could not be assessed because no pediatric evidence was identified. | | | | | |
| Problem | | Children and adolescents with refractory, chronic, or corticosteroid-dependent ITP may experience persistent bleeding risk, repeated hospital visits, prolonged corticosteroid exposure, and impaired quality of life. Younger children also face an important therapeutic gap because eltrombopag is not available for all pediatric age groups, making the evaluation of romiplostim particularly relevant. | | | |
| Benefits and harm | | Romiplostim was considered an alternative to eltrombopag, with no statistically significant difference in platelet response or serious adverse events, although clinically significant bleeding was higher in the indirect comparison. Rituximab showed superior platelet response compared with vincristine in the identified randomized trial. No evidence supported dapsone use in the pediatric population. Overall, the evidence suggested that rituximab and romiplostim are effective and reasonably safe options for selected pediatric patients, but uncertainty remains because of limited comparative evidence. | | | |
| Resource use | | The pediatric economic evaluation used a decision-tree model from the SUS perspective. Rituximab alone and rituximab combined with romiplostim were dominant compared with the current treatment sequence, with lower costs and higher QALYs. Romiplostim alone generated additional QALYs with an ICER of R$14,623 per QALY in the base case, although sensitivity analyses indicated uncertainty and possible higher-cost scenarios. Dapsone was not included in economic analyses because no pediatric efficacy and safety evidence was identified. | | | |
| Equity | | No formal equity assessment was reported. Nevertheless, incorporation of romiplostim may improve equity for younger children and for patients who do not respond to, cannot use, or are not eligible for other options. Incorporation of rituximab may also standardize access to a therapy already used in clinical practice but not consistently available through the SUS for this indication. | | | |
| Acceptability | | No formal equity assessment was reported. Nevertheless, incorporation of romiplostim may improve equity for younger children and for patients who do not respond to, cannot use, or are not eligible for other options. Incorporation of rituximab may also standardize access to a therapy already used in clinical practice but not consistently available through the SUS for this indication. | | | |
| Feasibility | | Rituximab and romiplostim were considered feasible for incorporation according to the CPG. Rituximab requires infusion capacity and monitoring for infusion reactions, infections, hepatitis B reactivation, and hypogammaglobulinemia. Romiplostim requires subcutaneous administration, platelet monitoring, and dose adjustment. Dapsone was not incorporated because of lack of pediatric evidence and instability in national supply. | | | |

CPG: Clinical Practice Guidelines; HTA: health technology assessment; SUS: Brazilian Unified Health System; TPO-RA: thrombopoietin receptor agonists; QALY: quality adjusted life years

References:

1. Brasil. Ministério da Saúde. Relatório de recomendação no 950 - Rituximabe, dapsona e romiplostim para o tratamento de indivíduos adultos com púrpura trombocitopênica idiopática primária refratária ou dependente de corticosteroide. Comissão Nacional de Incorporação de Tecnologias no Sistema Único de Saúde - CONITEC. https://www.gov.br/conitec/pt-br/midias/relatorios/relatorio-de-recomendacao-no-950-rituximabe-dapsona-e-romiplostim/view. Published 2026. Accessed May 4, 2026.

2. Brasil. Ministério da Saúde. Relatório de recomendação no 969 - Rituximabe, dapsona e romiplostim para o tratamento de crianças e adolescentes com púrpura trombocitopênica idiopática primária (PTI) refratária ou dependente de corticosteroide. Comissão Nacional de Incorporação de Tecnologias no Sistema Único de Saúde - CONITEC. https://www.gov.br/conitec/pt-br/midias/relatorios/2025/relatorio-de-recomendacao-no-969-rituximabe-e-o-romiplostim. Published 2025. Accessed May 4, 2026.

Table S4. Medicines and platelet transfusion strategies for emergency situations in children, adolescents, and adults with primary immune thrombocytopenia (ITP).

| **Product** | **Children and adolescents** | **Adults** |
| --- | --- | --- |
| Methylprednisolone | 15 mg/kg to 30 mg/kg for three days | 1 g/day for three days |
| Intravenous human immunoglobulin | 0.8 g/kg to 1 g/kg for one to two days; the dose may be repeated on the second day if platelet count remains below 50 x 10^3^/µL | 0.8 g/kg to 1 g/kg for one to two days; the dose may be repeated on the second day if platelet count remains below 50 x 10^3^/µL |
| Platelet transfusion with or without IVIg | A dose three times higher than usual is recommended because of rapid platelet destruction in ITP; 3 units for each 10 kg | A dose three times higher than usual is recommended because of rapid platelet destruction in ITP; 3 units for each 10 kg |

IVIg, intravenous immunoglobulin.
